# Influence of advanced life support response time on out-of-hospital cardiac arrest patient outcomes in Taipei

**DOI:** 10.1371/journal.pone.0266969

**Published:** 2022-04-14

**Authors:** Hsuan-An Chen, Shuo-Ting Hsu, Ming-Ju Hsieh, Shyh-Shyong Sim, Sheng-En Chu, Wen-Shuo Yang, Yu-Chun Chien, Yao-Cheng Wang, Bin-Chou Lee, Edward Pei-Chuan Huang, Hao-Yang Lin, Matthew Huei-Ming Ma, Wen-Chu Chiang, Jen-Tang Sun

**Affiliations:** 1 Department of Emergency Medicine, Far Eastern Memorial Hospital, New Taipei City, Taiwan; 2 Department of Emergency Medicine, National Taiwan University Hospital, College of Medicine, National Taiwan University, Taipei, Taiwan; 3 Emergency Medical Services Division, Taipei City Fire Department, Taipei City, Taiwan; 4 Emergency Medical Services Division, National Fire Agency, Ministry of the Interior, New Taipei City, Taiwan; 5 Department of Emergency Medicine, National Taiwan University Hospital, Taipei City Hospital, Zhongxiao Branch, Taipei, Taiwan; 6 Department of Emergency Medicine, National Taiwan University Hospital, Hsin-Chu Branch, Hsin-Chu City, Taiwan; 7 Department of Emergency Medicine, National Taiwan University Hospital, Yun-Lin Branch, Douliu City, Taiwan; 8 School of medicine, Tzu Chi University, Hualien City, Taiwan; Ohio State University, UNITED STATES

## Abstract

**Background:**

The association between out-of-hospital cardiac arrest patient survival and advanced life support response time remained controversial. We aimed to test the hypothesis that for adult, non-traumatic, out-of-hospital cardiac arrest patients, a shorter advanced life support response time is associated with a better chance of survival. We analyzed Utstein-based registry data on adult, non-traumatic, out-of-hospital cardiac arrest patients in Taipei from 2011 to 2015.

**Methods:**

Patients without complete data, witnessed by emergency medical technicians, or with response times of ≥ 15 minutes, were excluded. We used logistic regression with an exposure of advanced life support response time. Primary and secondary outcomes were survival to hospital discharge and favorable neurological outcomes (cerebral performance category ≤ 2), respectively. Subgroup analyses were based on presenting rhythms of out-of-hospital cardiac arrest, bystander cardiopulmonary resuscitation, and witness status.

**Results:**

A total of 4,278 cases were included in the final analysis. The median advanced life support response time was 9 minutes. For every minute delayed in advanced life support response time, the chance of survival to hospital discharge would reduce by 7% and chance of favorable neurological outcome by 9%. Subgroup analysis showed that a longer advanced life support response time was negatively associated with the chance of survival to hospital discharge among out-of-hospital cardiac arrest patients with shockable rhythm and pulse electrical activity groups.

**Conclusions:**

In non-traumatic, adult, out-of-hospital cardiac arrest patients in Taipei, a longer advanced life support response time was associated with declining odds of survival to hospital discharge and favorable neurologic outcomes, especially in patients presenting with shockable rhythm and pulse electrical activity.

## Introduction

Out-of-hospital cardiac arrest (OHCA) is a major disease worldwide, with a high mortality rate. Taiwan has approximately 9,815 cases of OHCA annually, with a survival rate of approximately 9.8% [1]. Time is one of the most important prognostic factors, especially the emergency medical services (EMS) response time. The EMS response time, the period of time from to the call to EMS arrival at the scene, is associated with the survival rate among OHCA patients [2]. Several recent reports showed that shorter EMS response times improve the survival rates and neurological outcomes in patients with OHCA [2–11].

The effect of advanced life support (ALS) treatments in prehospital settings remains controversial. Several studies reported that earlier ALS intervention is associated with increasing survival rate [12–16], while others revealed worsening or no benefit to survival [17–19]. Thus, the association between the survival of OHCA patients and ALS response time remains controversial and unclear. Grunau et al. and Kurz et al. [20, 21] demonstrated that early ALS arrival at the scene can reduce mortality, but Michelland et al. [22] showed no benefit to early ALS response time.

Regarding the EMS system in Taipei, previous studies showed that the intervention of ALS and the number of ALS personnel are associated with better outcomes in OHCA patients [11, 23–27]; however, the exact influence of ALS response time has not yet been examined. Thus, the objective of this study was to determine whether a shorter ALS response time was associated with an improved chance of survival in non-traumatic, adult, OHCA patients.

## Methods

### Study design and setting

We conducted a 5-year retrospective cohort study using prospectively collected Utstein-based registry data from the Taipei EMS to investigate the association between the response time of ALS care and OHCA patient outcomes. All methods were performed in accordance with the study protocol which was approved by the Institutional Review Board (IRB) of the National Taiwan University Hospital (201606007RIND). Informed consent was waived due to the anonymized database and retrospective nature of the study, which was also approved by IRB of the National Taiwan University Hospital. The preliminary version of the abstract had been published at the European Resuscitation Council annual conference 2019 in Slovenia before we develop it into a full-length article.

### Data source

The Utstein-based OHCA registry system from the Taipei EMS was initially developed for OHCA quality control [28]. The registry system data comprised dispatch records, modes and timing of prehospital care, patient demographics, arrest characteristics (witness status, bystander Cardiopulmonary Resuscitation (CPR), initial rhythm, and cardiac monitor), records on automated external defibrillator (AED), prehospital ALS treatment, and patient outcomes (survival to hospital discharge and neurologic status at discharge) [25]. The rate of missing data ranged from 0% to 2.6%.

### Taipei EMS system

Taipei City, a metropolitan area in Taiwan, Southeast Asia, covers 272 km^2^, with 2.6 million registered residents. The majority of the population is Taiwanese. Taipei City has an EMS system based on the fire service with a two-tiered response team, including a basic life support (BLS) team and an ALS team. Taipei City has 45 prehospital BLS stations with 1,279 emergency medical technician (EMT) intermediate staff, who have completed at least 320 hours of training; and four ALS stations with 141 EMT paramedics, who have completed at least 1280 hours of training and need to conduct OHCA re-training every year [25]. The BLS team is capable of performing defibrillation and placing a laryngeal mask airway (LMA). One BLS station has two BLS ambulances, and each ambulance is usually teamed with two EMTs, sometimes three, depending on the available human resources at the time of dispatching [25]. The ALS team providers are authorized to perform endotracheal tube intubation and intravenous injections of resuscitation medications, such as adrenaline, atropine, and amiodarone, as per protocol [28]. One ALS station has three ALS ambulances, each usually teamed with two paramedics.

There is a single central dispatch center in Taipei to handle all incoming EMS calls; all dispatchers are required to complete 40 hours of training on priority dispatch. The BLS team is the universal response for all dispatch calls. For cases that meet the ALS dispatch criteria, such as out-of-hospital cardiac arrest, foreign body airway obstruction, major trauma, consciousness change and chest pain judged by dispatchers through the calls, additional ALS teams are dispatched to the scene together with BLS teams. For an ALS case that occurs in an area close to an ALS squad, the nearby ALS team is the first response team to dispatch, and an additional ALS team, as opposed to a BLS, will be activated if available [25].

### Study population

We analyzed OHCA patients in Taipei from 1 January 2011 to 31 December 2015. The eligible patients were adults (aged ≥ 20 years) with non-traumatic OHCA who underwent resuscitation attempts by the ALS team. Patients without complete data, with OHCA witnessed by EMT, with traumatic OHCA, or a response time longer than 15 minutes were excluded. Patients were also excluded if they were not transported to the hospital due to obvious signs of death, such as rigor mortis, if the family requested a do-not-resuscitate (DNR), or if the patient had a pre-existing DNR. We then divided the study population (Any ALS) into two groups, ALS dispatched only (only ALS) and ALS and BLS dispatched (ALS+BLS). We did not differentiate the arrival sequence in the unit of ALS+BLS, because the time interval between both groups was close, and the BLS assessment might have been affected by the ALS team.

### Definition of exposures

The key exposures in our study were ALS response time, defined as the interval from first EMS dispatch call to first ALS team arriving at the scene by the centralized time of dispatch. Other characteristics included EMS response time, witnessed OHCA, bystander CPR, shockable rhythm, OHCA happening in public places, scene time, transporting to medical centers, injecting adrenaline prehospitally, injecting other medications prehospitally, such as atropine and amiodarone, and endotracheal tube intubation. EMS response time was defined as the period of time from to the call to EMS arrival at the scene (regardless of BLS or ALS team); shockable rhythm was defined as the heart rhythm showing pulseless ventricular tachycardia (pVT) or ventricular fibrillation (Vf) during resuscitation and scene time was defined as the portion of time between arrival of the ambulance on scene of the patient and when the ambulance departs the scene.

### Outcome measurement

The primary outcome was survival to hospital discharge. The secondary outcome was favorable neurological outcome, defined as cerebral performance category (CPC) level 1 and 2 [29], which is a key endpoint for several prominent clinical trials [30, 31] and a core recommended outcome measure for cardiac arrest registries [32].

### Statistical analysis

We used Excel (Microsoft, Redmond, WA, USA) to record data and SAS version 9.3 (SAS Institute, Cary, NC, USA) to analyze the data. The descriptive statistics for the population are presented as counts, percentages, or medians (interquartile range [IQR] Q1–Q3). We performed chi-squared or Fisher’s exact tests to assess the associations between the categorical variables and outcomes. For continuous variables, we conducted non-parametric Mann–Whitney rank sum tests for analyses. All variables previously determined to be associated with the outcomes were included in the multivariable logistic regression analysis to prevent overfitting. Odds ratios (ORs) and 95% confidence intervals (CIs) were calculated, and two-tailed p-values < 0.05 were considered statistically significant.

We conducted a subgroup analysis using the new Utstein template, with methods suggested by the International Liaison Committee on Resuscitation (ILCOR) in 2014, to explore the effect of ALS response time among different subgroups of patients with OHCA. For this analysis, we stratified the data based on presenting rhythms of OHCA, including shockable rhythm (pVT/Vf), pulseless electrical activity (PEA), and asystole [33]. We also analyzed a subgroup of patients with witnessed OHCA and patients with bystander CPR. Known Utstein covariates, including age, sex, witnessed OHCA, bystander CPR, shockable rhythm, total EMT numbers, and EMS response time were adjusted. We further separated the ALS response time into categorical variables (< 8 minutes, 8–11 minutes, ≥ 11 minutes) by patient numbers to explore the cut-off value of the ALS response interval. A restricted cubic spline model was performed on the total study population and subgroup analysis to visualize the association between ALS response time and survival to hospital discharge.

## Results

### Study population

**Fig 1:**
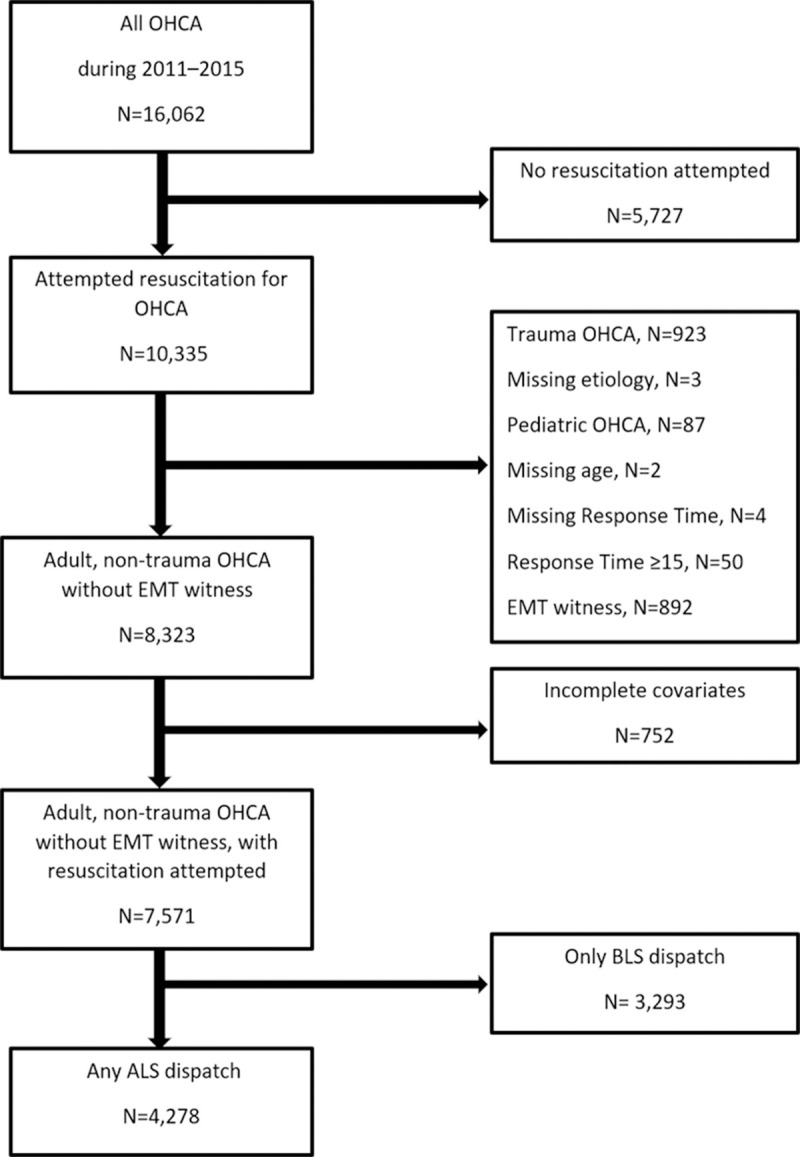
Study population.

Of the 16,062 OHCA cases treated between 2010 and 2015, 7,571 cases were adult, non-trauma OHCA without EMT witness with resuscitation attempted and 4,278 cases with ALS dispatch were included in the final analysis. (Fig 1) The proportion of ALS dispatch cases were 56.5% (4278/7571). Patient characteristics are listed in Table 1. The median ALS response time was 9 minutes (IQR 7–12), the median response time of the first ambulance was 5 minutes (IQR 4–6), and the median scene time was 15 minutes (IQR 13–18). A total of 1366 (31.93%) patients received an adrenaline injection, and 789 (18.44%) patients received endotracheal tube intubation. A total of 993 (23.21%) patients achieved a return of spontaneous circulation (ROSC), 287 (6.71%) survived to discharge, and 126 (2.95%) had a favorable clinical outcome (CPC ≤ 2) at discharge.

**Table 1:** Demographic data and outcomes of enrolled out-of-hospital cardiac arrest patients stratified based on disp^a^tch group.

	Any ALS (n = 4,278)	Only ALS (n = 661)	ALS+BLS (n = 3,617)	p-value^a^
**Patient characteristics and received treatments**				
Age, years (median [Q1–Q3])	78 (64, 86)	77 (63, 85)	78 (64, 86)	0.017
Male, number (percent)	2652 (61.99%)	422 (63.84%)	2230 (61.65%)	0.29
Witness, number (percent)	1248 (29.17%)	238 (36.01%)	1010 (27.92%)	< .0001
Bystander CPR, number (percent)	1669 (39.01%)	220 (33.28%)	1449 (40.06%)	0.0010
Shockable rhythm, number (percent)	436 (10.19%)	88 (13.31%)	348 (9.62%)	0.0039
Public, number (percent)	326 (7.62%)	92 (13.92%)	234 (6.47%)	< .0001
ALS Response time, minutes (median [Q1–Q3])	9 (7, 12)	6 (4, 7)	10 (8, 12)	< .0001
EMS Response time, minutes (median [Q1–Q3])	5 (4, 6)	6 (4, 7)	5 (4, 6)	< .0001
Scene time, minutes (median [Q1–Q3])	15 (13, 18)	16 (13, 19)	15 (12, 17)	< .0001
Medical center, number (percent)	2125 (49.67%)	360 (54.46%)	1765 (48.8%)	0.0074
Adrenaline, number (percent)	1366 (31.93%)	280 (42.36%)	1086 (30.02%)	< .0001
Atropine/amiodarone/others, number (percent)	57 (1.33%)	17 (2.57%)	40 (1.11%)	0.0025
Endotracheal tube, number (percent)	789 (18.44%)	195 (29.5%)	594 (16.42%)	< .0001
**Survival status, number (percent)**				
Any ROSC	1149 (26.86%)	227 (34.34%)	922 (25.49%)	< .0001
Sustained ROSC	993 (23.21%)	199 (30.11%)	794 (21.95%)	< .0001
Survival to hospital discharge	287 (6.71%)	69 (10.44%)	218 (6.03%)	< .0001
CPC≦ 2 at discharge	126 (2.95%)	36 (5.45%)	90 (2.49%)	< .0001

^a^ Group (only ALS) compared to group (ALS+BLS)

ALS: advanced life support. BLS: basic life support. CPR: cardiopulmonary resuscitation. CPC: cerebral performance category. ROSC: return of spontaneous circulation.

### Outcomes

Table 2 shows the adjusted odds ratio (aOR) for survival to hospital discharge and favorable clinical outcomes in different groups based on ALS response time (per minute). For every minute of delayed ALS response time, the likelihood of survival to hospital discharge would reduce by 7% (aOR 0.93; 95% CI: 0.89–0.97) in all OHCA patients. Further, for every minute of delayed ALS response time, there was a reduction of 9% (aOR, 0.91; 95% CI: 0.85–0.97) chance of favorable neurologic outcome (CPC ≤ 2) at discharge. Univariate analysis of each group in Table 2 were showed in S1 and S2 Tables. In the subgroup analysis, the chance of survival to hospital discharge decreased by 9% (aOR 0.91; 95% CI: 0.85–0.97) in the shockable rhythm (pVT/Vf) group, decreased by 9% (aOR 0.91; 95% CI: 0.85–0.98) in the PEA rhythm group, and had no significant benefit in the asystole rhythm group. The chance of a favorable neurological outcome at discharge decreased by 9% (aOR 0.91; 95% CI: 0.85–0.97) every minute in general ALS resuscitation, by 11% (aOR 0.89; 95% CI: 0.81–0.96) in shockable rhythm, and 12% (aOR 0.88; 95% CI: 0.78–0.996) in PEA. Similarly, there were no significant benefits in the asystole rhythm group.

**Table 2:** Multivariable logistic regression of survival to hospital discharge and neurological outcome with predictor of each minute of ALS response time.

	Any ALS		Only ALS		ALS+BLS	
	N	Adjusted OR	N	Adjusted OR	N	Adjusted OR (95% CI)
	(STHD/total)	(95% CI)	(STHD/total)	(95% CI)	(STHD/total)	
**Survival to hospital discharge (N = 287)**						
All OHCA patients	287/4,278	0.93 (0.89–0.97)^a^*	69/661	0.94 (0.82–1.07)^c^	218/3,617	0.94 (0.90–0.99)^a^*
Shockable rhythm (pVT/Vf)	122/436	0.91 (0.85–0.97)^b^*	31/88	0.79 (0.61–1.04)^d^	91/348	0.91 (0.85–0.99)^b^*
Non-shockable rhythm	165/3,842	0.94 (0.89–0.99)^b^*	38/573	0.97 (0.83–1.13)^d^	127/3,269	0.96 (0.91–1.01)^b^
PEA	87/800	0.91 (0.85–0.98)^b^*	20/154	0.87 (0.66–1.14)^d^	67/646	0.91 (0.83–0.99)^b^*
Asystole	78/3,018	0.98 (0.91–1.05)^b^	18/415	1.05 (0.86–1.27)^d^	60/2,603	1.01 (0.94–1.08)^b^
**CPC1–CPC2 (N = 126)**	**(CPC1-2/total)**		**(CPC1-2/total)**		**(CPC1-2/total)**	
All OHCA patients	126/4,278	0.91 (0.85–0.97)^a^*	36/661	0.92 (0.76–1.12)^c^	90/3,617	0.93 (0.87–0.997)^a^*
Shockable rhythm (pVT/Vf)	80/436	0.89 (0.81–0.96)^b^*	28/88	0.86 (0.67–1.11)^d^	52/348	0.92 (0.83–1.01)^b^
Non-shockable rhythm	46/3,842	0.94 (0.85–1.03)^b^	8/573	0.93 (0.66–1.30)^d^	38/3,269	0.95 (0.86–1.05)^b^
PEA	33/800	0.88 (0.78–0.996)^b^*	7/154	0.92 (0.62–1.37)^d^	26/646	0.88 (0.77–1.01)^b^
Asystole	13/3,018	1.06 (0.96–1.17)^b^	1/415	NA^e^	12/2,603	1.04 (0.93–1.17)^b^

^a^: Adjusted by EMS response time, total EMT, age, sex, witness, bystander CPR, shockable rhythm.

^b^: Adjusted by EMS response time, total EMT, age, sex, witness, bystander CPR.

^c^: Adjusted by total EMT, age, sex, witness, bystander CPR, shockable rhythm.

^d^: Adjusted by total EMT, age, sex, witness, bystander CPR.

^e^: All observations have the same response.

*: P-value<0.05

ALS: advanced life support. BLS: basic life support. CPC: cerebral performance category. OHCA: out-of-hospital cardiac arrest. pVT: pulseless ventricular tachycardia. PEA: pulseless electrical activity. VF: ventricular fibrillation

Table 3 further demonstrates the aOR of survival to hospital discharge and favorable neurological outcome after separating ALS response time to tertiles. We found that compared to the group with ALS response time of less than 8 minutes, in the group with ALS response time over 11 minutes every minute of delayed ALS response time would decrease by 40% (aOR 0.6; 95% CI: 0.43–0.84) the chance of survival to hospital discharge, as well as favorable neurologic outcome (aOR 0.59; 95% CI: 0.35–0.97). We found a similar result in the group of witnessed OHCA patients and the group receiving bystander CPR. Favorable neurological outcome was not statistically significant in the bystander CPR group.

**Table 3:** Subgroups of multivariable logistic regression of survival to hospital discharge and neurological outcome in ALS response time interval (tertile).

Adjusted OR (95% CI) of 4 subgroups	Total N	N	ALS response time <8 minutes	N	ALS response time 8–11 minutes	N	ALS response time ≧ 11 minutes
**Survival to hospital discharge**							
Any ALS^a^	4,278	1383	Reference	1344	0.86 (0.62, 1.17)	1550	0.60 (0.43, 0.84)*
Witnessed status							
Witnessed^b^	1,248	460	Reference	373	0.78 (0.52, 1.19)	415	0.56 (0.36, 0.86)*
Non-witnessed^b^	3,030	923	Reference	971	0.95 (0.58, 1.55)	1135	0.68 (0.40, 1.15)
Bystander CPR							
Bystander CPR^c^	1,669	541	Reference	563	0.81 (0.52, 1.26)	565	0.42 (0.25, 0.70)*
Non-bystander CPR^c^	2,609	842	Reference	781	0.88 (0.56, 1.38)	985	0.82 (0.53, 1.28)
**CPC1–CPC2**							
Any ALS^a^	4,278	1,383	Reference	1,344	0.88 (0.55, 1.41)	1,550	0.59 (0.35, 0.97)*
Witnessed status							
Witnessed^b^	1,248	460	Reference	373	0.69 (0.38, 1.23)	415	0.47 (0.25, 0.87)*
Non-witnessed^b^	3,030	923	Reference	971	1.37 (0.61, 3.08)	1,135	0.94 (0.39, 2.26)
Bystander CPR							
Bystander CPR^c^	1,669	541	Reference	563	1.14 (0.61, 2.14)	565	0.66 (0.33, 1.33)
Non-bystander CPR^c^	2,609	842	Reference	781	0.64 (0.30, 1.34)	985	0.54 (0.26, 1.13)

^a^: Adjusted by EMS response time, total EMT, age, sex, witness, bystander CPR, shockable rhythm

^b^: Adjusted by EMS response time, total EMT, age, sex, bystander CPR, shockable rhythm

^c^: Adjusted by EMS response time, total EMT, age, sex, witness, shockable rhythm

*: P value<0.05

ALS: advanced life support. CPR: cardiopulmonary resuscitation. CPC: cerebral performance category

**Fig 2:**
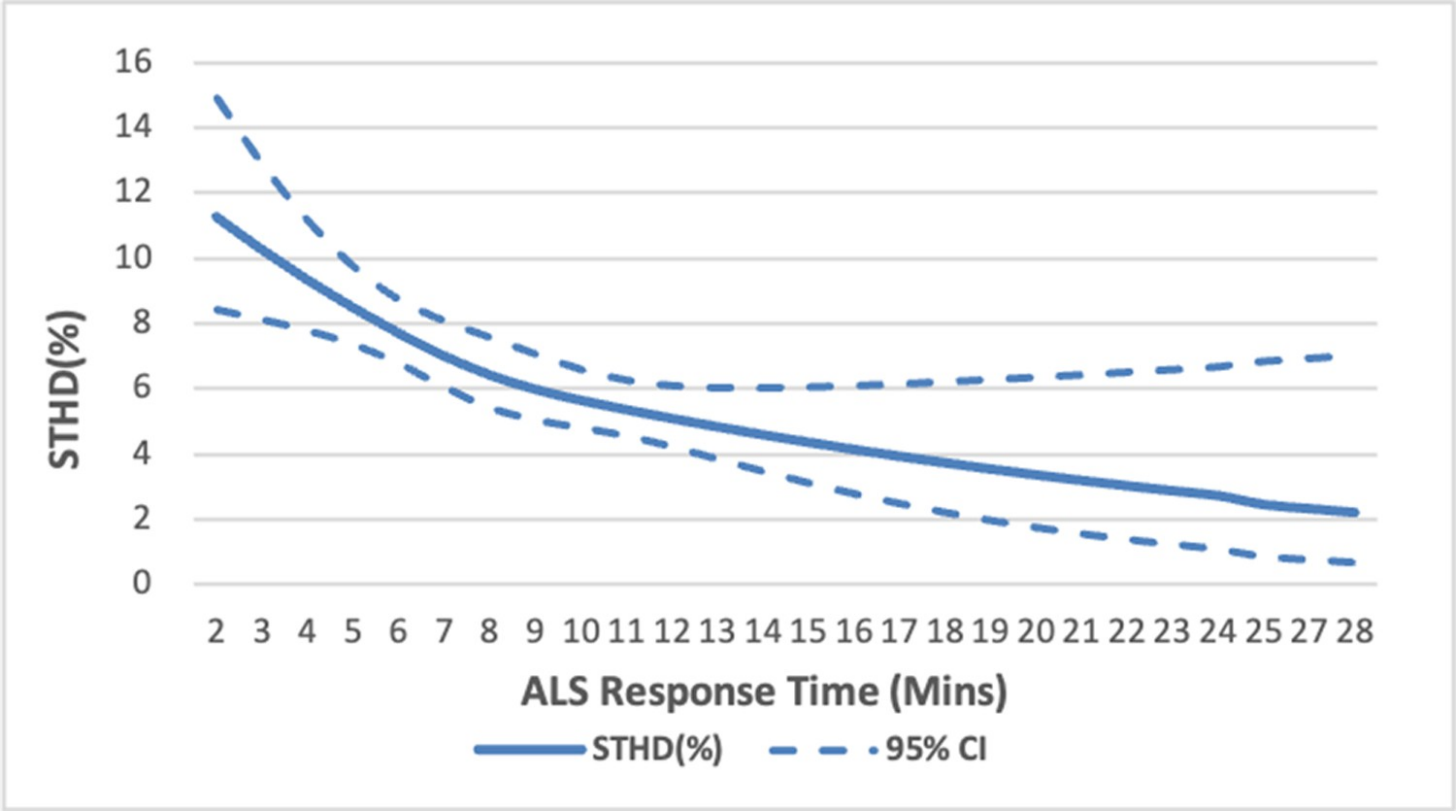
Spline curves for the outcomes of survival to hospital discharge (with 95% confidence intervals), as a function of ALS response time. Abbreviations: ALS = advanced life support. STHD = survival to hospital discharge.

**Fig 3:**
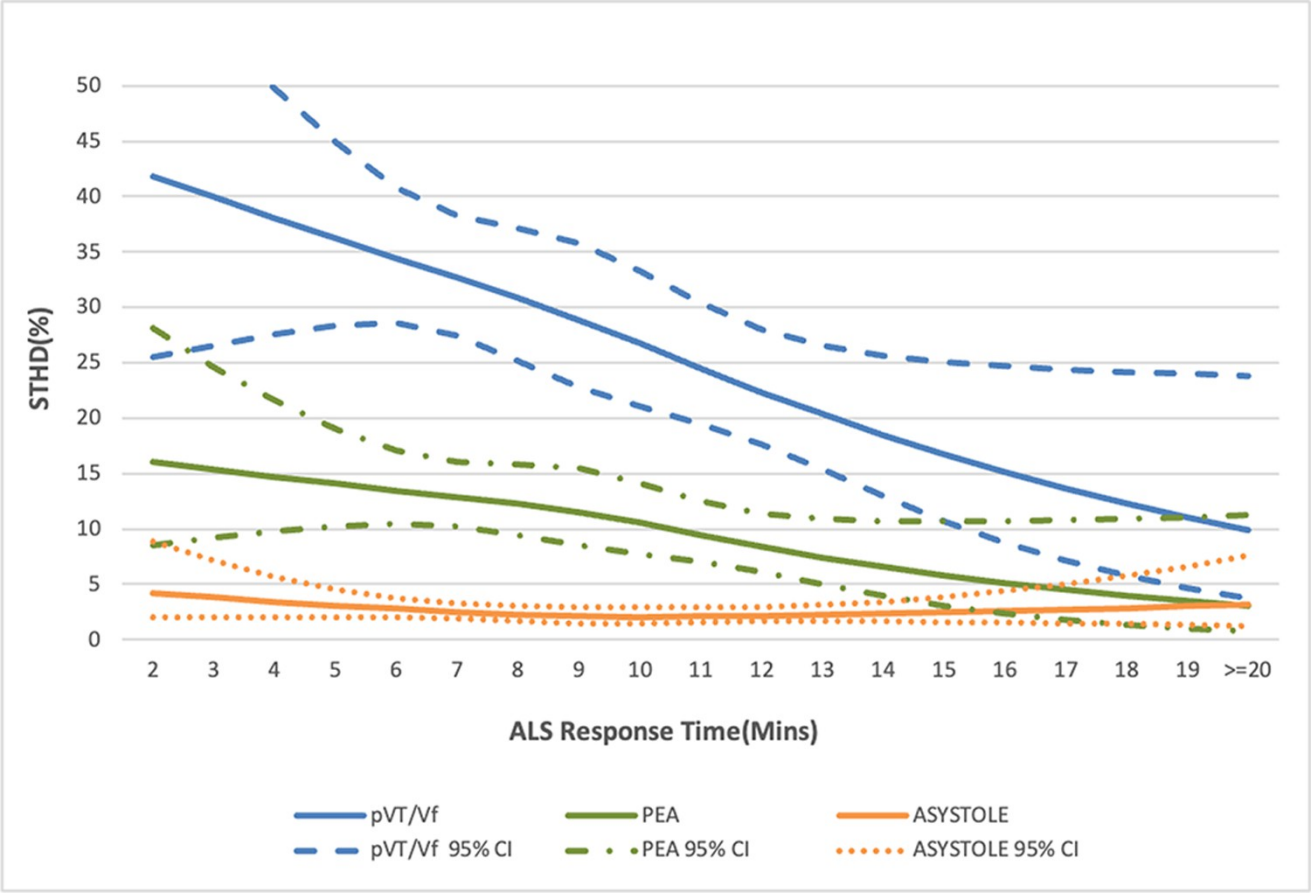
Restricted cubic spline curve for the survival to hospital discharge (%) and ALS response time with subgroups according to initial cardiac rhythm of the patients. Abbreviations: ALS = advanced life support. STHD = survival to hospital discharge. pVT = pulseless ventricular tachycardia. PEA = pulseless electrical activity. VF = ventricular fibrillation.

The restricted cubic spline curve in Fig 2 demonstrates the estimated trend of decrease in the rate of survival to hospital discharge as ALS response time increases, which has a sharper step-wise decline in the chance of survival with increasing time intervals. The decline stabilized at approximately 8 minutes. Fig 3 further demonstrates the estimated results of the subgroup divided by initial rhythm (pVT/Vf, PEA, asystole). The group of shockable rhythm and PEA also demonstrates a trend of decline in the chance of survival with increasing ALS response time intervals.

## Discussion

In this large observational study in Taipei, we found that every minute of delay in ALS response time was associated with a 7% reduction in survival to hospital discharge and a 9% reduction in favorable neurological outcome in adult, non-traumatic, OHCA patients. A swift response by ALS not only benefited all OHCA patients, but also significantly benefited the subgroups of patients initially presenting shockable rhythms or PEA. An ALS response time of less than 8 minutes was associated with better outcomes in OHCA patients, while over 11 minutes was associated with a diminished chance of survival. We previously announced the preliminary results of this study [11], which was also consistent to our final results.

Our findings are similar to those of Grunau et al. [20], with new information. Their study demonstrates that the rate of survival to hospital discharge decreases by 3% for each minute of delayed ALS response time (aOR 0.97, 95% CI: 0.96–0.98). The results are consistent with ours, except for different decrease rates, which could be attributed to a higher proportion of patients with shockable rhythm and a higher proportion of bystander CPR rates. We further observed a benefit of early ALS arrival in the subgroup of patients with PEA. In our subgroup analysis, every minute of delayed ALS response time decreased the survival to hospital discharge by 9% (aOR 0.91, 95% CI: 0.85–0.98) in the PEA group. The deteriorating rate was almost the same as in the group with shockable rhythm. Survival outcomes in the group with non-shockable rhythm are lower than those with shockable rhythm in previous studies [34–36]. However, several studies support the observed trend that OHCA patients with a first recorded rhythm of PEA have significantly higher survival rates than those presenting with asystole [37–40]. “Pseudo-PEA”, or “pulseless with a rhythm with echocardiographic motion (PREM)”, which refers to patients with PEA but a beating heart under ultrasound, could be the cause. Studies reveal that patients with PREM had higher survival rates than those with PEA without echocardiographic motion; aggressive ALS treatment may increase their survival [41–43].

There are several reasonable explanations for early ALS team response time improving the rate of survival to hospital discharge and neurological outcome among OHCA patients. ALS treatment includes airway management and drug administration. Network meta-analysis of randomized control trials reveal that ET tube placement and supraglottic airway (SGA) do increase the rate of ROSC compared to BVM [44]; the success rate of intubation greatly influenced the results. In one randomized clinical trial from Taipei EMS, among patients with OHCA, the initial airway management with ETI by ALS was associated with a higher probability of prehospital ROSC compared with SGA, especially among the subgroups of non-shockable rhythm, nonpublic collapse, arrested witnessed, call to airway time less than 14 minutes, and age 77 years or older, indicating that the shorter ALS response time may be related to a better chance of prehospital ROSC [45]. Chiang et al. [23] also found that successful out-of-hospital intubation with OHCA patients increased the odds of sustained ROSC, survival to hospital discharge, and favorable neurological outcome compared to BVM. With regard to adrenaline administration, Perkin et al. [16] reported that a prehospital adrenaline injection increases survival in OHCA patients by 30 days and further analysis reveals that an early injection of adrenaline can increase the probability of ROSC and survival to 30 days [46, 47]. Several studies also demonstrate that a delayed adrenaline injection might decrease the rate of survival to hospital discharge in OHCA patients and favorable neurological outcomes in non-shockable OHCA patients [48–50]. Furthermore, previous studies in the Taipei area show that the ALS team can enhance outcomes in OHCA patients. Ma et al. [24] found that an ALS team can improve ROSC and survival to admission. Sun et al. [25] found that an on-scene EMT–paramedics ratio > 50% is associated with improved survival to hospital discharge for OHCA cases, especially for those with witnessed, non-shockable rhythm. In addition, the awareness of OHCA management among the EMT is increasing and the ALS crews are more experienced, good at teamwork, and enthusiastic [24]. Paramedics with a 5-year OHCA case volume of ≥ 15 are significantly associated with ROSC [51], and in Taipei, one paramedic treated almost all of the 10 OHCA patients in 1 year [24]. Several studies have demonstrated that more experienced paramedics can improve the outcomes of OHCA patients [52, 53].

Some studies have opposed ALS treatment. The OPALS study compared the before and after of implementation of ALS paramedics into EMS in Canada. The results revealed no improvement in the rate of survival to hospital discharge and functional outcome in OHCA patients after ALS intervention [54]. Although by far the best evidence, the before and after study design and the lack of familiarity and clinical experience of newly-trained ALS crews could partially explain the results. The change in ALS treatment over the years may also improve patient outcomes. Sanghavi et al. [55] compared the BLS and ALS treatment in non-traumatic OHCA patients using a nationally representative sample of traditional Medicare beneficiaries from nonrural counties in the United States, and found that the ALS group was associated with poor neurological and survival outcomes. Using Medicare beneficiaries’ data could result in reporting bias and an overestimation of mortality [56]. A lack of at-scene data and ALS arrival time may also influence the results, as our study demonstrated that the time of ALS intervention was an important factor affecting outcomes. The use of national data could increase external validity while decreasing internal validity because the EMS systems, dispatchers, and paramedics are distinct from others in different states or areas. Michelland et al. [22] compared the early and late ALS arrivals in non-traumatic OHCA patients using propensity score matches in France, and found that early ALS intervention was associated with a lower rate of ROSC and neurological outcome at discharge after matching. The average arrival time of the ALS team in the early ALS group was 15 minutes in Michelland’s study, while our study revealed that the effect of ALS intervention diminished if the ALS response time was longer than 11 minutes. Grunau et al. [20] also demonstrated that the optimal cut-off time for ALS intervention was 10 minutes. In addition, the BLS team arrival time was shorter in the delayed ALS group than in the early ALS group (10 minutes vs. 13 minutes), which could have also affected the results.

### Limitations

Our study had several limitations. First, this was a retrospective observational study. While selection bias inherent to our study design cannot be eliminated, we believe it was mitigated by our population-based approach. While we adjusted for common OHCA confounders, additional unmeasured or unmeasurable confounders may have been present, such as in-hospital care, which may have influenced OHCA survival [31, 57]. Second, we did not compare the difference between the ALS and only-BLS groups as the response time gap was close, and the BLS-only group may have included a higher proportion of patients who achieved rapid ROSC with defibrillation. Third, our study subjects were identified from the metropolitan regions of one Taiwanese city; causality, logistical, political, and ethical complexities in different settings may have limited the external validity. Furthermore, we examined the effect of ALS care on OHCA; other disease states were not discussed.

## Conclusion

In non-traumatic, adult, OHCA patients in Taipei, a longer ALS response time was associated with worse odds of survival to hospital discharge and favorable neurological outcomes, especially in patients presenting with shockable rhythm and PEA.

Our study further suggests the optimal ALS response time for the EMS system.

## Supporting information

S1 Table Univariate logistic regression of survival to hospital discharge in each group. ALS: advanced life support. BLS: basic life support. CPR: cardiopulmonary resuscitation. EMS: emergency medical service. EMT: emergency medical technician. OHCA: out-of-hospital cardiac arrest. OR: odds ratio. pVT: pulseless ventricular tachycardia. PEA: pulseless electrical activity. STHD: survival to hospital discharge. VF: ventricular fibrillation.
(DOCX)

S2 Table Univariate logistic regression of CPC1–CPC2 in each group. ALS: advanced life support. BLS: basic life support. CPC: cerebral performance category. CPR: cardiopulmonary resuscitation. EMS: emergency medical service. EMT: emergency medical technician. OHCA: out-of-hospital cardiac arrest. OR: odds ratio. pVT: pulseless ventricular tachycardia. PEA: pulseless electrical activity. VF: ventricular fibrillation. ^a^: cannot performed due to N = 1 in group CPC1-2.
(DOCX)
