# Supplementary material for: Influence of advanced life support response time on out-of-hospital cardiac arrest patient outcomes in Taipei
Source: PLoS One. 2022 Apr 14;17(4):e0266969. doi: 10.1371/journal.pone.0266969 (PMC9009650; doi:10.1371/journal.pone.0266969)
Supplement: S1 Table — ALS: advanced life support. BLS: basic life support. CPR: cardiopulmonary resuscitation. EMS: emergency medical service. EMT: emergency medical technician. OHCA: out-of-hospital cardiac arrest. OR: odds ratio. pVT: pulseless ventricular tachycardia. PEA: pulseless electrical activity. STHD: survival to hospital discharge. VF: ventricular fibrillation. (DOCX) [file pone.0266969.s001.docx]

**Influence of advanced life support response time on out-of-hospital cardiac arrest patient outcomes in Taipei**

**Supporting information**

S1 Table. Univariate logistic regression of survival to hospital discharge in each group.

|  | **Any ALS** | | **Only ALS** | | **ALS+BLS** | |
| --- | --- | --- | --- | --- | --- | --- |
| **Survival to hospital discharge** | **OR (95%CI)** | **P value** | **OR (95%CI)** | **P value** | **OR (95%CI)** | **P value** |
| **All OHCA patients** | **(STHD/total N = 287/4,278)** | | **(STHD/total N = 69/661)** | | **(STHD/total N = 218/3,617)** | |
| ALS Response Time | 0.92 (0.89-0.96) | <0.001 | 0.95 (0.85-1.07) | 0.423 | 0.94 (0.90-0.98) | 0.005 |
| EMS Response Time | 0.98 (0.92-1.05) | 0.596 |  |  | 0.97 (0.89-1.05) | 0.376 |
| Total EMT | 0.91 (0.77-1.07) | 0.233 | 1.32 (0.99-1.76) | 0.063 | 1.05 (0.82-1.35) | 0.710 |
| Shockable Rhythm (pVT/Vf) | 8.68 (6.67-11.24) | <0.001 | 7.66 (4.43-13.24) | <0.001 | 8.76 (6.51-11.80) | <0.001 |
| Age | 0.97 (0.967-0.98) | <0.001 | 0.97 (0.96-0.99) | <0.001 | 0.97 (0.966-0.98) | <0.001 |
| Sex | 1.37 (1.06-1.77) | 0.017 | 1.69 (0.96-2.96) | 0.068 | 1.28 (0.96-1.71) | 0.096 |
| Witness | 4.46 (3.48-5.72) | <0.001 | 4.17 (2.45-7.07) | <0.001 | 4.41 (3.32-5.84) | <0.001 |
| Bystander CPR | 1.57 (1.22-1.98) | <0.001 | 1.74 (1.05-2.88) | 0.032 | 1.57 (1.19-2.06) | 0.001 |
| **Shockable rhythm (pVT/Vf)** | **(STHD/total N = 122/436)** | | **(STHD/total N = 31/88)** | | **(STHD/total N = 91/348)** | |
| ALS Response Time | 0.90 (0.85-0.96) | <0.001 | 0.85 (0.68-1.08) | 0.186 | 0.91 (0.85-0.97) | 0.007 |
| EMS Response Time | 0.88 (0.78-0.98) | 0.024 |  |  | 0.86 (0.75-0.99) | 0.031 |
| Total EMT | 0.95 (0.72-1.26) | 0.740 | 1.63 (0.98-2.69) | 0.059 | 0.85 (0.53-1.37) | 0.51 |
| Age | 0.97 (0.96-0.99) | <0.001 | 1.00 (0.97-1.03) | 0.938 | 0.97 (0.95-0.98) | <0.001 |
| Sex | 1.23 (0.71-2.14) | 0.0465 | 1.10 (0.30-4.00) | 0.883 | 1.22 (0.66-2.26) | 0.532 |
| Witness | 1.95 (1.24-3.06) | 0.004 | 1.81 (0.69-4.74) | 0.230 | 1.95 (1.17-3.26) | 0.011 |
| Bystander CPR | 1.51 (0.99-2.30) | 0.056 | 1.93 (0.80-4.69) | 0.145 | 1.45 (0.90-2.35) | 0.132 |
| **Non-shockable rhythm** | **(STHD/total N = 165/3,842)** | | **(STHD/total N = 38/573)** | | **(STHD/total N = 127/3,269)** | |
| ALS Response Time | 0.94 (0.90-0.98) | 0.007 | 0.98 (0.84-1.14) | 0.785 | 0.96 (0.91-1.01) | 0.101 |
| EMS Response Time | 1.01 (0.93-1.10) | 0.777 |  |  | 1.00 (0.90-1.11) | 0.967 |
| Total EMT | 0.91 (0.74-1.13) | 0.395 | 1.14 (0.77-1.70) | 0.514 | 1.14 (0.83-1.57) | 0.417 |
| Age | 0.99 (0.98-1.003) | 0.167 | 0.99 (0.97-1.01) | 0.221 | 1.00 (0.98-1.01) | 0.370 |
| Sex | 0.91 (0.66-1.25) | 0.554 | 1.13 (0.57-2.24) | 0.718 | 0.85 (0.60-1.22) | 0.377 |
| Witness | 3.83 (2.79-5.25) | <0.001 | 3.69 (1.88-7.26) | <0.001 | 3.76 (2.63-5.38) | <0.001 |
| Bystander CPR | 1.26 (0.92-1.72) | 0.155 | 1.29 (0.65-2.55) | 0.472 | 1.30 (0.91-1.85) | 0.156 |
| **PEA** | **(STHD/total N = 87/800)** | | **(STHD/total N = 20/154)** | | **(STHD/total N = 67/646)** | |
| ALS Response Time | 0.91 (0.85-0.98) | 0.008 | 0.88 (0.69-1.12) | 0.296 | 0.91 (0.84-0.99) | 0.021 |
| EMS Response Time | 0.97 (0.86-1.10) | 0.601 |  |  | 0.99 (0.86-1.15) | 0.897 |
| Total EMT | 1.03 (0.76-1.38) | 0.871 | 1.11 (0.63-1.96) | 0.722 | 1.25 (0.77-2.03) | 0.363 |
| Age | 0.99 (0.98-1.01) | 0.191 | 0.99 (0.96-1.02) | 0.558 | 0.99 (0.98-1.01) | 0.269 |
| Sex | 0.73 (0.46-1.14) | 0.165 | 0.84 (0.32-2.19) | 0.717 | 0.70 (0.42-1.16) | 0.165 |
| Witness | 2.66 (1.66-4.27) | <0.001 | 3.38 (1.16-9.83) | 0.025 | 2.48 (1.46-4.22) | 0.001 |
| Bystander CPR | 1.05 (0.67-1.66) | 0.82 | 1.08 (0.39-3.03) | 0.880 | 1.08 (0.65-1.80) | 0.761 |
| **Asystole** | **(STHD/total N = 78/3018)** | | **(STHD/total N = 18/415)** | | **(STHD/total N = 60/2,603)** | |
| ALS Response Time | 0.98 (0.92-1.04) | 0.511 | 1.07 (0.88-1.30) | 0.503 | 1.01 (0.94-1.08) | 0.843 |
| EMS Response Time | 1.07 (0.95-1.20) | 0.257 |  |  | 1.03 (0.89-1.19) | 0.715 |
| Total EMT | 0.85 (0.63-1.16) | 0.309 | 1.17 (0.66-2.07) | 0.589 | 1.08 (0.67-1.72) | 0.762 |
| Age | 0.99 (0.98-1.01) | 0.316 | 0.99 (0.96-1.02) | 0.321 | 1.00 (0.98-1.01) | 0.522 |
| Sex | 1.02 (0.64-1.64) | 0,923 | 1.38 (0.51-3.75) | 0.529 | 0.93 (0.55-1.59) | 0.797 |
| Witness | 2.69 (1.67-4.33) | <0.001 | 2.65 (1.02-6.92) | 0.046 | 2.60 (1.50-4.51) | 0.001 |
| Bystander CPR | 1.30 (0.82-2.08) | 0.265 | 1.68 (0.65-4.36) | 0.285 | 1.24 (0.73-2.13) | 0.424 |

ALS: advanced life support. BLS: basic life support. CPR: cardiopulmonary resuscitation. EMS: emergency medical service. EMT: emergency medical technician. OHCA: out-of-hospital cardiac arrest. OR: odds ratio. pVT: pulseless ventricular tachycardia. PEA: pulseless electrical activity. STHD: survival to hospital discharge. VF: ventricular fibrillation.
