# Supplementary material for: Influence of advanced life support response time on out-of-hospital cardiac arrest patient outcomes in Taipei
Source: PLoS One. 2022 Apr 14;17(4):e0266969. doi: 10.1371/journal.pone.0266969 (PMC9009650; doi:10.1371/journal.pone.0266969)
Supplement: S2 Table — ALS: advanced life support. BLS: basic life support. CPC: cerebral performance category. CPR: cardiopulmonary resuscitation. EMS: emergency medical service. EMT: emergency medical technician. OHCA: out-of-hospital cardiac arrest. OR: odds ratio. pVT: pulseless ventricular tachycardia. PEA: pulseless electrical activity. VF: ventricular fibrillation. a: cannot performed due to N = 1 in group CPC1-2. (DOCX) [file pone.0266969.s002.docx]

**Influence of advanced life support response time on out-of-hospital cardiac arrest patient outcomes in Taipei**

**Supporting information**

S2 Table. Univariate logistic regression of CPC1–CPC2 in each group.

|  | **Any ALS** | | **Only ALS** | | **ALS+BLS** | | |
| --- | --- | --- | --- | --- | --- | --- | --- |
| **CPC1–CPC2** | **OR(95%CI)** | **P value** | **OR(95%CI)** | **P value** | **OR(95%CI)** | **P value** | |
| **All OHCA patients** | **(CPC1-2/total N = 126/4,278)** | | **(CPC1-2/total N = 36/661)** | | **(CPC1-2/total N = 90/3,617)** | | |
| ALS Response Time | 0.91 (0.86-0.96) | <0.001 | 0.97 (0.83-1.14) | 0.738 | 0.93 (0.87-0.99) | | 0.029 |
| EMS Response Time | 1.01 (0.91-1.10) | 0.923 |  |  | 0.97 (0.86-1.10) | | 0.664 |
| Total EMT | 0.84 (0.63-1.06) | 0.132 | 1.15 (0.77-1.72) | 0.481 | 1.16 (0.79-1.70) | | 0.439 |
| Shockable Rhythm (pVT/Vf) | 18.54 (12.70-27.08) | <0.001 | 32.96 (14.38-75.55) | <0.001 | 14.94 (9.67-23.07) | | <0.001 |
| Age | 0.96 (0.95-0.97) | <0.001 | 0.96 (0.94-0.98) | <0.001 | 0.96 (0.95-0.97) | | <0.001 |
| Sex | 2.10 (1.37-3.18) | <0.001 | 2.45 (1.06-5.67) | 0.037 | 1.95 (1.20-3.17) | | 0.007 |
| Witness | 5.13 (3.26-7.48) | <0.001 | 4.40 (2.12-9.11) | <0.001 | 5.16 (3.32-8.01) | | <0.001 |
| Bystander CPR | 2.06 (1.44-2.95) | <0.001 | 2.36 (1.20-4.63) | 0.013 | 2.09 (1.37-3.19) | | <0.001 |
| **Shockable rhythm (pVT/Vf)** | **(CPC1-2/total N = 80/436)** | | **(CPC1-2/total N = 28/88)** | | **(CPC1-2/total N = 52/348)** | | |
| ALS Response Time | 0.88 (0.82-0.95) | <0.001 | 0.95 (0.76-1.19) | 0.626 | 0.91 (0.84-0.996) | 0.0402 | |
| EMS Response Time | 0.95 (0.83-1.07) | 0.386 |  |  | 0.89 (0.75-1.05) | 0.166 | |
| Total EMT | 0.72 (0.53-0.98) | 0.035 | 1.07 (0.65-1.77) | 0.787 | 0.96 (0.53-1.74) | 0.901 | |
| Age | 0.97 (0.96-0.99) | <0.001 | 0.99 (0.96-1.02) | 0.474 | 0.97 (0.95-0.99) | 0.001 | |
| Sex | 1.22 (0.64-2.33) | 0.554 | 0.92 (0.25-3.37) | 0.904 | 1.22 (0.56-2.63) | 0.622 | |
| Witness | 1.81 (1.06-3.06) | 0.028 | 1.87 (0.69-5.08) | 0.223 | 1.69 (0.90-3.18) | 0.104 | |
| Bystander CPR | 1.64 (1.002-2.69) | 0.049 | 2.67 (1.06-6.72) | 0.037 | 1.48 (0.81-2.69) | 0.203 | |
| **Non-shockable rhythm** | **(STHD/total N = 46/3,842)** | | **(STHD/total N = 8/573)** | | **(STHD/total N = 38/3,269)** | | |
| ALS Response Time | 0.95 (0.87-1.03) | 0.214 | 0.95 (0.68-1.32) | 0.740 | 0.95 (0.86-1.04) | 0.267 | |
| EMS Response Time | 1.01 (0.86-1.18) | 0.903 |  |  | 1.02 (0.86-1.22) | 0.804 | |
| Total EMT | 1.17 (0.78-1.77) | 0.446 | 1.24 (0.54-2.81) | 0.615 | 1.41 (0.79-2.50) | 0.241 | |
| Age | 0.99 (0.97-1.01) | 0.190 | 0.98 (0.94-1.02) | 0.375 | 0.99 (0.97-1.01) | 0.298 | |
| Sex | 1.27 (0.69-2.33) | 0.451 | 1.10 (0.26-4.63) | 0.902 | 1.30 (0.66-2.56) | 0.442 | |
| Witness | 4.21 (2.33-7.61) | <0.001 | 2.21 (0.55-8.94) | 0.266 | 4.80 (2.49-9.25) | <0.001 | |
| Bystander CPR | 1.66 (0.93-2.97) | 0.088 | 0.72 (0.14-3.60) | 0.688 | 1.96 (1.03-3.73) | 0.040 | |
| **PEA** | **(CPC1-2/total N = 33/800)** | | **(CPC1-2/total N = 7/154)** | | **(CPC1-2/total N = 26/646)** | | |
| ALS Response Time | 0.90 (0.81-1.00) | 0.0495 | 0.94 (0.64-1.38) | 0.761 | 0.87 (0.76-0.99) | 0.037 | |
| EMS Response Time | 0.95 (0.78-1.16) | 0.629 |  |  | 0.95 (0.75-1.20) | 0.660 | |
| Total EMT | 1.14 (0.71-1.84) | 0.590 | 1.20 (0.49-2.94) | 0.694 | 1.38 (0.66-2.90) | 0.400 | |
| Age | 0.98 (0.96-1.003) | 0.09 | 0.99 (0.94-1.03) | 0.537 | 0.98 (0.96-1.01) | 0.117 | |
| Sex | 1.36 (0.64-2.90) | 0.426 | 1.45 (0.27-7.74) | 0.663 | 1.34 (0.57-3.12) | 0.504 | |
| Witness | 1.8 (0.88-3.66) | 0.108 | 0.72 (0.16-3.33) | 0.674 | 2.31 (1.01-5.26) | 0.046 | |
| Bystander CPR | 1.61 (0.80-3.23) | 0.181 | 1.00 (0.19-5.36) | 1 | 1.85 (0.84-4.10) | 0.129 | |
| **Asystole** | **(CPC1-2/total N = 13/3,018)** | | **(CPC1-2/total N = 1/415)^a^** | | **(CPC1-2/total N = 12/2,603)** | | |
| ALS Response Time | 1.06 (0.99-1.13) | 0.1187 | NA | NA | 1.05 (0.96-1.14) | 0.2822 | |
| EMS Response Time | 1.17 (0.90-1.52) | 0.2525 | NA | NA | 1.22 (0.93-1.60) | 0.1498 | |
| Total EMT | 2.04 (0.83-5.03) | 0.1241 | NA | NA | 1.70 (0.60-4.80) | 0.3211 | |
| Age | 1.00 (0.96-1.04) | 0.8756 | NA | NA | 1.00 (0.97-1.04) | 0.8704 | |
| Sex | 0.84 (0.26-2.75) | 0.7683 | NA | NA | 0.84 (0.26-2.76) | 0.7733 | |
| Witness | 3.39 (1.03-11.15) | 0.0443 | NA | NA | 3.55 (1.08-11.67) | 0.0371 | |
| Bystander CPR | 1.42 (0.43-4.66) | 0.5645 | NA | NA | 1.38 (0.42-4.53) | 0.5975 | |

ALS: advanced life support. BLS: basic life support. CPC: cerebral performance category. CPR: cardiopulmonary resuscitation. EMS: emergency medical service. EMT: emergency medical technician. OHCA: out-of-hospital cardiac arrest. OR: odds ratio. pVT: pulseless ventricular tachycardia. PEA: pulseless electrical activity. VF: ventricular fibrillation

^a^: cannot performed due to N=1 in group CPC1-2.
